# Establishment of a Bacterial Expression System and Immunoassay Platform for the Major Capsid Protein of HcRNAV, a Dinoflagellate-Infecting RNA Virus

**DOI:** 10.1264/jsme2.ME12046

**Published:** 2012-10-05

**Authors:** Kei Wada, Kei Kimura, Akifumi Hasegawa, Keiichi Fukuyama, Keizo Nagasaki

**Affiliations:** 1 Department of Biological Sciences, Graduate School of Science, Osaka University, Toyonaka, Osaka 560–0043, Japan; 2 National Research Institute of Fisheries and Environment of Inland Sea, Fisheries Research Agency, 2–17–5 Maruishi, Hatsukaichi, Hiroshima 739–0452, Japan

**Keywords:** bacterial expression, *Heterocapsa circularisquama* RNA virus, major capsid protein, polyclonal antibody

## Abstract

HcRNAV is a small icosahedral virus that infects the shellfish-killing marine dinoflagellate *Heterocapsa circularisquama*, which harbors a dicistronic linear single-stranded RNA (ssRNA) genome *ca.* 4.4 kb in length. Its major capsid protein (MCP) gene sequence is not expressed by various strains of *Escherichia coli*, possibly because of a codon usage problem. To solve this problem, a chemically modified (*i.e., de novo* synthesized) gene was designed and cloned into the pCold-GST expression vector, and transformed into *E. coli* strain C41 (DE3), in which codon usage was universally optimized to efficiently express the polypeptide having the viral MCP amino acid sequence. The bacterially expressed protein, which was purified after a procedure involving denaturation and refolding, successfully formed virus-like particles that significantly resembled native HcRNAV particles. The purified, denatured protein was used as an antigen to immunize rabbits, and the resulting antiserum was shown to be strongly reactive to not only the bacterially expressed recombinant protein, but also to native HcRNAV MCP by Western blotting and dot immunoassays, respectively. These results indicate that an antiserum recognizing native HcRNAV MCP was successfully obtained using bacterially expressed HcRNAV MCP as the antigen.

To date, eight RNA viruses infecting marine eukaryotic microorganisms have been isolated and characterized to a different extent. Among them, seven are single-stranded RNA (ssRNA) viruses: HaRNAV infects the noxious bloom-forming raphidophyte *Heterosigma akashiwo* (Raphidophyceae) (10, 28); RsetRNAV (17, 26), CtenRNAV (27), CsfrRNAV (32) and AglaRNAV (33) are diatom-infecting viruses that infect *Rhizosolenia setigera*, *Chaetoceros tenuissimus*, *C. socialis*, and *Asterionellopsis glacialis*, respectively; HcRNAV infects the bivalve-killing bloom-forming dinoflagellate *Heterocapsa circularisquama* (15, 16, 18, 31); SssRNAV (recently approved as “*Aurantiochytrium* single-stranded RNA virus” by ICTV) infects the marine fungoid protist *Schizochytrium* sp. (Labyrinthula, Thraustochytriaceae) (29, 30); additionally, the other is a double-stranded RNA (dsRNA) virus (MpRNAV) that infects the cosmopolitan phytoplankton *Micromonas pusilla* (1, 4). Although detailed genomic analyses of these viruses have been performed, few studies have been undertaken to elucidate the detailed mechanism of their adsorption and infection of their specific host.

HcRNAV, which infects the noxious bloom-forming alga *H. circularisquama*, is the first and only dinoflagellate-infecting RNA virus grown in culture. The HcRNAV genome is a linear ssRNA, *ca.* 4.4 kb in length, and harbors two open reading frames (ORFs) (16); the upstream ORF (ORF1) encodes a replication polyprotein and the downstream ORF (ORF2) codes for a single major capsid protein (MCP). A recent cryo-electron microscopy study on HcRNAV particles revealed that the virus has a diameter of 34 nm and *T*=3 symmetry, and consists of 180 quasi-equivalent monomers (13). For dsDNA algal viruses, some notable studies on the entry process of the virus have been reported, including studies on the entry process of *Chlorella* virus (5, 12, 19, 24) and a phaeovirus (*Ectocarpus fasciculatus* virus) (11). In contrast, little is known about the mechanisms underlying the adsorption and infection of ssRNA algal viruses. This study was designed to establish a bacterial expression system for HcRNAV MCP and to prepare a polyclonal antibody targeting it by using the expressed MCP as an antigen, which is expected to be a promising tool to analyze the host-virus crosstalking process. Here, we have described the procedure to establish a bacterial expression system and an immunoassay platform for HcRNAV MCP as well as its promising availability.

## Materials and Methods

### Virion purification

The virus was collected from the HcRNAV-infected *H. circularisquama* culture using the method described by Tomaru *et al.* (31). Briefly, 450 mL of exponentially growing *H. circularisquama* strain HU9433-P was inoculated with 3 mL fresh suspension of HcRNAV strain 34 (16, 31) (HcRNAV34; ca. 10^7^ infectious units mL^−1^). The host cells were lysed and then sequentially filtered through 8.0-, 0.8-, and 0.2-μm filters to remove cell debris. Polyethylene glycol was added to the filtrate to obtain a final concentration of 10% (w/v), and the mixture was stored in the dark overnight at 4°C; the suspension was centrifuged at 57,000×*g* for 90 min. The viral pellet was then washed with phosphate buffer (10 mM Na_2_HPO_4_ and 10 mM KH_2_PO_4_ in distilled water) and centrifuged again at 217,000×*g* for 4 h at 4°C. The collected virus particles were resuspended in 500 μL phosphate buffer.

### Extraction of HcRNAV genomic ssRNA and cloning of the authentic MCP cDNA

Genomic ssRNA was purified from HcRNAV34 particles using the High Pure Viral RNA kit (Roche Applied Science, Penzberg, Germany) and reverse-transcribed with random oligo primers and PrimeScript Reverse Transcriptase (Takara, Otsu, Japan). The procedures for ssRNA isolation and reverse transcription (RT)-PCR were in accordance with the manufacturers’ protocols. A pair of the following primers was designed on the basis of the previously reported HcRNAV34 genomic sequence (NCBI Reference Sequence: NC_007518.1): 5′-CAT ATG ACC CGT CCC CTA GCT C-3′ and 5′-GGA TCC TCG AGC TCA AGC AGC CAT CAA TGC TGG C-3′, which were used to amplify the MCP gene (ORF2) by PCR. PCR amplification was performed using PrimeSTAR Max DNA Polymerase (Takara). The PCR cycle was as follows: 1 cycle of denaturation at 98°C (20 s), 30 cycles of (denaturation at 98°C [10 s], annealing at 55°C [5 s], and extension at 72°C [65 s]), followed by an additional extension at 72°C (65 s). The PCR product was initially subcloned into pTA2 (TOYOBO, Osaka, Japan), and the sequence was confirmed by resequencing. For expression in *E. coli*, the gene fragment encoding authentic ORF2 was ligated into the pET-15b and pET-21a expression vectors (Merck Biosciences, Darmstadt, Germany). For the expression in yeast, the gene fragment encoding N-terminal His-tagged authentic ORF2 derived from the pET-15b-*orf2* vector was cloned into pHIL-D2 and pPIC9 vectors (Invitrogen, San Diego, CA, USA), and the vector constructs were integrated into the genome of *Pichia pastoris*. Expression of His-tagged ORF2 was also attempted using insect cells, where the isolation and transfection of bacmid DNA into Sf21 cells was carried out according to the Bac-to-Bac baculovirus expression system manual (Invitrogen).

### Cloning of the synthesized ORF2 gene into an expression vector

To chemically (*de novo*) synthesize the MCP gene of HcRNAV34, the nucleotide sequence was designed according to the deduced amino acid sequence of HcRNAV34 MCP to optimize codon usage for the expression in *E. coli* (Invitrogen); here, the original deduction was performed on the basis of universal codon usage. The artificially synthesized gene was cloned into the *Nde*I/*Bam*HI sites of the pCold-GST expression vector to produce an N-terminus His-tagged GST-fusion MCP (His-GST-MCP), where the cleavage site by human rhinovirus 3C (HRV3C) protease was introduced between His-GST and MCP (the pCold-GST vector was kindly provided by Dr. C. Kojima, Institute for Protein Research, Osaka University).

### Overproduction and purification of recombinant MCP

The pCold-His_6_-GST-ORF2 expression vector was transformed into *E. coli* strain C41 (DE3). The transformant was grown at 37°C in 5.4 L (900 mL × 6) of liquid Terrific broth containing ampicillin (50 μg mL^−1^) to an optical density of 0.6 at 600 nm. At this stage, expression of His-GST-MCP was induced by lowering the temperature from 37°C to 15°C, and then adding 1 mM isopropyl-β-d-thiogalactopyranoside. After induction, the transformant was cultured at 15°C for an additional 24 h. The cells were harvested, suspended in 50 mM Tris-HCl (pH 7.8), and disrupted by sonication. The insoluble fraction was resuspended in buffer *A* (50 mM Tris-HCl [pH 7.8], 8 M urea, 20 mM imidazole, and 300 mM NaCl). This suspension was mixed with nickel-affinity resin (Nacalai Tesque, Kyoto, Japan) and incubated for 1 h. The lysate/resin mixture was washed with buffer *A*, and protein elution was carried out with a modified buffer *A* (50 mM Tris-HCl [pH 7.8], 8 M urea, 300 mM imidazole, and 300 mM NaCl). The fractions containing His-GST-MCP were collected, concentrated with a Vivaspin filter (GE Healthcare, Milwaukee, WI, USA), diluted to 1:10 with a FoldIt Screen kit (Hampton Research, Laguna Niguel, CA, USA) buffer condition 8 (55 mM MES [pH 6.5], 264 mM NaCl, 0.055% [w/v] PEG 3350, 1.1 mM EDTA, and 550 mM l-arginine), and incubated overnight at 4°C. After determination of the protein concentration by the Bradford assay, His-GST-MCP was cleaved with 10 U of HRV3C protease (Accelagen, San Diego, CA, USA) per milligram of protein, and incubated overnight at 4°C. The cleaved proteins were separated by SDS-PAGE using a 12.5% (w/v) polyacrylamide gel, and the zinc-imidazole stained bands (20) corresponding to MCP (*ca.* 35-kDa band) were excised from the gels. MCP in the gels was extracted using the Electro-Eluter (Bio-Rad Laboratories, Hercules, CA, USA), according to the manufacturer’s protocol.

### Refolding and self-assembly of recombinant MCP

Denatured recombinant MCP of HcRNAV in SDS-PAGE running buffer was refolded by a dilution method. Briefly, 20 μL purified MCP solution obtained by gel extraction (approx. 2 mg mL^−1^) was diluted into 180 μL of FoldIt Screen kit buffer condition 8 containing the various amphipathic solvents at 2 M (final conc.), such as 2-propanol, 2-methyl-2,4-pentandiol, 2-butanol, and 1-butanol, which have different partition coefficients (LogP), and incubated overnight at 4°C. The refolding mixture was subjected to size exclusion chromatography on a Superose 6 (GE Healthcare) column equipped with the ÄKTA explorer 10S system. The peak fraction of 280 nm absorbance was collected and analyzed by an H-7650 transmission electron microscope (Hitachi, Tokyo, Japan) at an acceleration voltage of 80 kV.

### Preparation of polyclonal antibody for MCP

The purified recombinant MCP in SDS-PAGE running buffer was mixed with Freund’s complete adjuvant (Sigma-Aldrich, St. Louis, MO, USA) and intradermally injected into rabbits at a dose of 0.1 mg protein per rabbit. Two weeks after the first inoculation, hypodermic inoculations of 0.1 mg protein per rabbit with Freund’s incomplete adjuvant were performed weekly for 6 weeks, and then the rabbits were exsanguinated to collect the antisera. Finally, the polyclonal antibody (designated as “ABH-1”) was stored at −80°C or at −30°C until use.

### ELISA

TBS (20 mM Tris-HCl [pH 7.4], 2.7 mM KCl, and 100 mM NaCl) containing 1 μg purified recombinant MCP was placed in each well of a 96-well microtiter plate and incubated for 18 h at 4°C. After removing the antigen fluid, the wells were blocked with TBS containing 2% BSA for 2 h at room temperature. After washing the wells three times with TBS containing 0.05% Tween-20, the serial dilution solution (1/[5n]) of the polyclonal antibody was added to each well, followed by peroxidase-conjugated goat anti-rabbit IgG antibody (Medical & Biological Laboratories, Nagoya, Japan) for 2 h at room temperature. Finally, the remaining peroxidase activity was determined using 3,3′,5,5′-tetramethylbenzidine (TMB) (Medical & Biological Laboratories) as a substrate. The results were monitored spectrophotometrically at an optical density of 450 nm.

### Dot blot analysis

Protein solutions (33 μg mL^−1^) were spotted onto an Immobilon-P Transfer Membrane (Millipore, Bedford, MA, USA) at 2 μL dot^−1^, and the membrane was blocked with 5% (w/v) skim milk in TBST (50 mM Tris-HCl [pH 7.8], 0.1% [v/v] Tween 20, and 150 mM NaCl) for 1 h; subsequently, it was washed with TBST. The anti-MCP polyclonal antibody was diluted 1:40,000 in TBST, and the membrane was incubated with diluted anti-MCP antibody for 1 h. The membrane was then washed with TBST, incubated for 1 h with anti-rabbit IgG [H+L] (Goat) conjugated to horseradish peroxidase (Nacalai Tesque) and diluted to 1:40,000 in TBST, washed again, developed with an ECL Plus Western Blotting Detection System (GE Healthcare), and then, the resultant membrane was exposed to Black & White Instant Film (Fujifilm, Tokyo, Japan) using an ECL Mini-camera (GE Healthcare).

### Western blotting

Uninfected and HcRNAV34-infected cells of *H. circularisquama* (2 d post inoculation [d pi]) were collected by centrifugation at 750×*g* for 10 min. HcRNAV34 particles were collected by the methods described above. The sample pellets were mashed in SDS sample buffer (50 mM Tris hydroxymethyl aminomethane-HCl, 2% [w/w] SDS, 10% [v/v] glycerol, 6% [v/v] 2-mercaptoethanol, 0.002% [w/v] bromophenol blue, pH 6.8) using a micro-homogenizer and boiled for 5 min. The samples were then electrophoresed at 200 V for 30 min using a Mini-PROTEAN Cell system with a Mini-PROTEAN TGX Precast Gel 12% (Bio-Rad) according to the method of Laemmli (9), and the gel was stained with Coomassie Brilliant Blue R-250 (CBB). The molecular mass was estimated using Precision Standards (Bio-Rad). The gels were blotted onto polyvinylidene difluoride (PVDF) membranes (Seque-Blot PVDF Membrane, Bio-Rad) using the Mini-TRANSBLOT System (Bio-Rad). The membrane was stained with CBB to capture the protein staining pattern. The membrane was decolorized with 100% methanol, washed in TBS (20 mM Tris-HCl, 150 mM NaCl, pH 7.5) several times, and then immunostained with the polyclonal antibody ABH-1 (diluted 1:10,000 in modified TBST containing 0.05% [v/v] Tween-20) using the ProtoBlot II AP Systems with Stabilized Substrate (Promega, Madison, USA), according to the manufacturer’s protocol. Another PVDF membrane treated with TBST served as the control. The immunostaining pattern of the resultant membrane was captured.

### Immunofluorescence microscopy

Immunofluorescence observation was performed according to the method of Motomura and Hishinuma (14). To fix the intact *H. circularisquama* cells and virus-infected cells (2 d pi), each cell suspension was mixed with an equal volume of fixation buffer (2% [w/v] glutaraldehyde and 3% [w/v] paraformaldehyde in PHEM buffer [60 mM Pipes, 25 mM Hepes, 10 mM EGTA, 2 mM MgCl_2_, pH 7.4] with 3% NaCl) for 30 min at 4°C. Each sample was then placed on a poly-l-lysine-coated cover glass for 10 min to allow the cells to attach. After washing with PBS (137 mM NaCl, 2.7 mM KCl, 4.9 mM Na_2_HPO_4_, 1.5 mM KH_2_PO_4_, pH 7.4), the cells on the cover glass were treated with 5% (v/v) Triton-X100 in PBS, 0.1% (w/v) NaBH_4_ in PBS for 20 min, and then rinsed with PBS several times. Samples were incubated in blocking solution (2.5% [w/v] nonfat milk, 5% [w/v] normal goat serum, and 0.05% [w/v] NaN_3_ in PBS) for 30 min at 37°C. After blocking, they were incubated at 20°C overnight with the polyclonal antibody ABH-1 diluted 1:1,000 in PBS. After washing in PBS, the samples were incubated with fluorescein isothiocyanate (FITC)-conjugated goat anti-rabbit IgG (1:1,000; Kirkegaad & Perry Laboratories, Gaithersburg, USA) for 1 h at 37°C, followed by washing with PBS. The immunostained samples were then mounted in Mowiol 4-88 mounting medium containing 0.2% (w/v) p-phenylenediamine, and observed using an Olympus epifluorescence microscope (BX-51, Olympus, Tokyo, Japan). The cell images were captured with the Digital Sight DS-Fi1 system (Nikon, Tokyo, Japan). Cells immunostained with a monoclonal anti-alpha-tubulin antibody (Clone DM1A, Sigma-Aldrich) and anti-mouse IgG antibody conjugated with FITC (Rockland, Gilbertsville, USA) served as the positive control.

## Results and Discussion

### Overproduction and purification of recombinant MCP

**Fig. 1:**
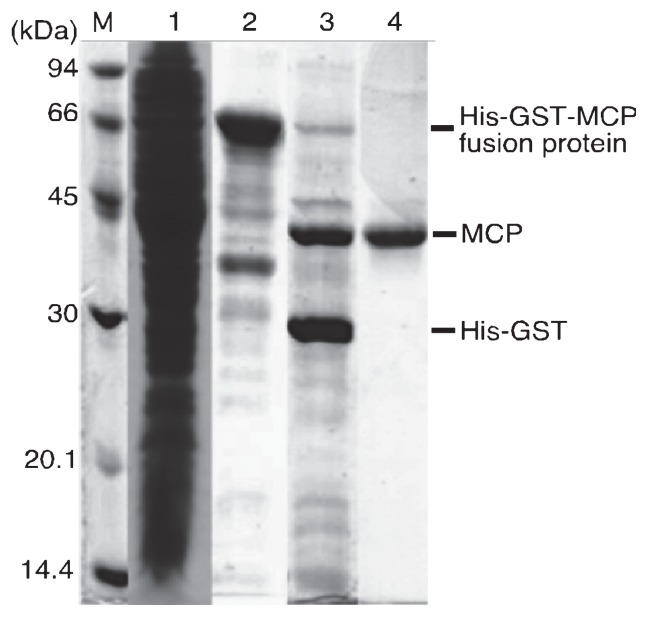
SDS-PAGE analysis of HcRNAV samples from each purification step. Gels were stained with Coomassie Brilliant Blue R-250. M, molecular weight markers; lane 1, insoluble fraction after sonication; lane 2, nickel-affinity purified His-GST-MCP fusion protein under denaturing conditions; lane 3, His-GST-MCP fusion protein treated with HRV3C protease; lane 4, recombinant MCP purified by gel extraction. This figure was made by combining each lane of independently performed SDS-PAGE.

Although we intended to establish an overproduction system of recombinant HcRNAV MCP using the authentic nucleotide sequence in *E. coli*, yeast and insect cells, all initial trials were unsuccessful (Fig. S1). Generally, the proportion of each codon used for a given amino acid varies widely between organisms; 15%–20% of the codons were revealed to correspond to rare codons that confound heterologous protein expression. Here, the genetic code used in the host dinoflagellate cell was assumed to be “universal”, because the results of amino acid sequencing due to Edman degradation well coincided in the previous analysis of the HcRNAV genome (16). Hence, an artificial sequence to optimize codon usage for the expression in *E. coli* cells was designed on the basis of the deduced amino acid sequence of HcRNAV MCP, and synthesized *de novo* (Fig. S2). Consequently, only when the pCold-His_6_-GST-ORF2 expression vector was transformed into the *E. coli* strain C41 (DE3) was His-GST-MCP was successfully overexpressed in the insoluble fraction. The expressed His-GST-MCP was solubilized with the denaturant and purified with nickel-affinity resin under denaturing conditions (Fig. 1). The purified His-GST-MCP was successfully refolded by the dilution method, and we successfully obtained His-GST-MCP in the soluble fraction. His-GST-MCP was cleaved by HRV3C protease; the subsequent SDS-PAGE gel showed CBB-stained major bands estimated to be at ~35 kDa and ~30 kDa (Fig. 1), which were consistent with the molecular masses of MCP and His-GST, respectively, calculated on the basis of their amino acid sequence. To collect pure MCP separated from the His-GST region, a variety of purification methods such as ion-exchange chromatography, hydrophobic interaction chromatography, and size-exclusion chromatography were applied; however, the MCP and His-GST region were eluted in the same fractions for all these chromatography methods, suggesting the possibility of aggregate formation. The mixture had significantly low migration in a native-PAGE gel and showed only a smeared band near the top of the gel (data not shown), which was consistent with aggregate formation. Hence, we decided to purify MCP by using gel extraction under denaturing conditions; MCP was separated from His-GST by SDS-PAGE and was electrically extracted from the gel. As a result, the gel extraction method provided a target protein with high purity (Fig. 1); a sufficient amount (~2 mg) of recombinant HcRNAV MCP, separated from the His-GST region, was successfully obtained by repetitive extractions. This protein was then used as an immunogen for the production of antiserum.

### Self-assembly of recombinant MCP

**Fig. 2:**
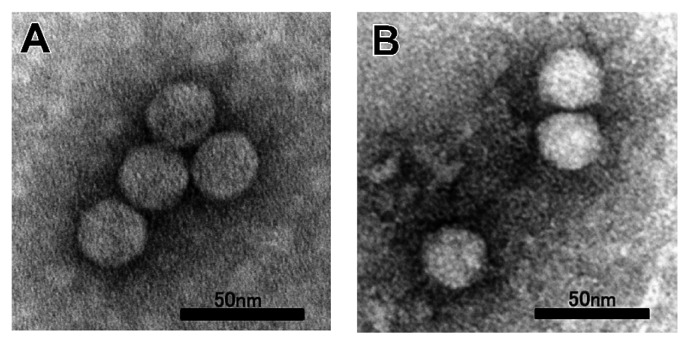
TEM images of negatively-stained VLPs formed by self-assembly of the recombinant MCP (A) and native HcRNAV particles (B).

In further experiments, reconstitution of the icosahedral architecture by using purified recombinant MCP was attempted to evaluate the quality of the recombinant MCP. Under the present experimental conditions, the main hindrance to inhibition of reconstitution was removal of the SDS bound to MCP, because the recombinant MCP was purified by extraction from SDS-PAGE gel. Some amphipathic solvents have been reported to be useful to remove tightly bound SDS from SDS-denatured proteins (8). Hence, we tried combining the buffer used in the dilution-refolding method (FoldIt Screen kit) with amphipathic solvents. When 2-propanol was used as the amphipathic solvent in the refolding reagent, the chromatogram from the subsequent size-exclusion chromatography column showed a peak corresponding to macromolecular formation (*i.e.*, formation of virus-like particles [VLPs]), although the several peaks in the small molecular size region were also detected (Fig. S3). Addition of other amphipathic solvents was not successful. The morphology of the particles in the peak fraction was examined by electron microscopy; spherical VLPs were clearly observed (Fig. 2). The VLPs and native HcRNAV particles were almost indistinguishable in size and shape: VLPs were icosahedral and *ca.* 30 nm in diameter, similar to native HcRNAV (Fig. 2). The formation of VLPs strongly suggested that the MCP protein expressed in this study, which was designed by the universal codon usage, has a similar nature to authentic MCP protein; however, the reconstruction efficiency of the VLPs was so low (<1%; efficiency was estimated based on the total protein amount) that further characterization is still needed to elucidate the functional features or VLPs.

### Reactivity of anti-MCP polyclonal antibody

**Fig. 3:**
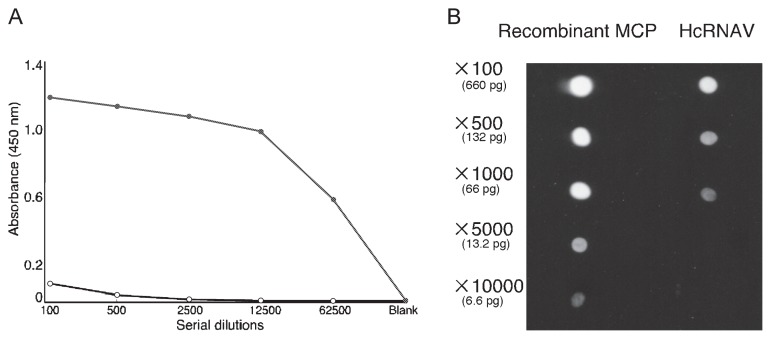
A. Reactivity of the anti-MCP polyclonal antibody ABH-1 to recombinant HcRNAV MCP measured by an enzyme-linked immunosorbent assay. The absorbance values of the anti-MCP antibody and pre-immunized antibody are indicated by closed and open circles, respectively. B. Reactivity of the anti-MCP polyclonal antibody ABH-1 measured by the dot blot assay. To compare reactivity, the proteins (recombinant HcRNAV MCP solution and intact HcRNAV suspension) were serially diluted.

Initially, we validated the reactivity of the anti-MCP polyclonal antibody (ABH-1) by ELISA. ABH-1 effectively recognized recombinant MCP, while serum from an unimmunized rabbit was scarcely reactive (Fig. 3A). The anti-MCP antibody produced in this study had a high titer against recombinant MCP; its titer (50% binding titer) was estimated to be 1:50,000. By dot blot analysis using intact HcRNAV particles as the target antigen, ABH-1 was shown to successfully recognize intact HcRNAV under non-denaturing conditions (Fig. 3B). Based on the protein concentration of the original antigen samples and the dilution rate at which positive reactivity was detected, the detection limit of ABH-1 for recombinant MCP and intact HcRNAV particles was estimated at ~13 pg and ~66 pg, respectively. Considering that ABH-1 was obtained using completely denatured MCP as an immunogen, it may be reasonable that ABH-1 is less reactive with HcRNAV retaining native MCP with intact 3D/4D stuctures.

### Specificity of ABH-1

**Fig. 4:**
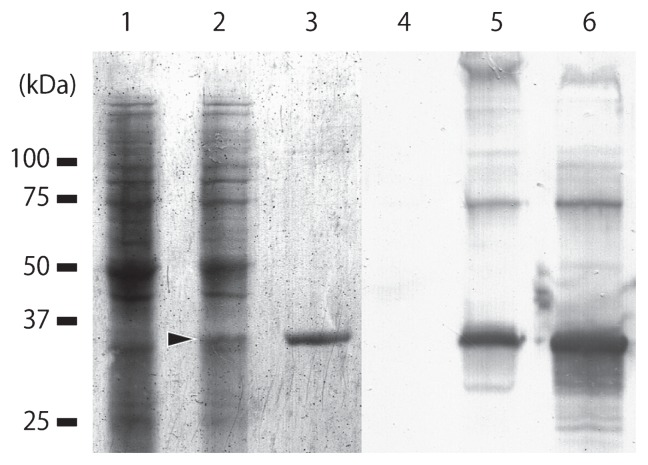
Western blotting analysis of the anti-MCP polyclonal antibody ABH-1 against native HcRNAV protein. Lanes 1–3: Coomassie Brilliant Blue-stained PVDF membrane from the trans-blotted SDS-PAGE gel. lanes 4–6, PVDF membrane after immunostaining using ABH-1 antibodies. Lane 1 and 4, whole cell proteins of uninfected *H. circularisquama*; lane 2 and 5, whole cell proteins of virus-infected *H. circularisquama*; lane 3 and 6, whole proteins of native HcRNAV particles. Arrowhead in lane 2 indicates a ~35-kDa band specifically observed in HcRNAV-infected cells.

To check the specificity of ABH-1, Western blotting analysis was performed. A clear band of ~35-kDa molecular mass (Fig. 4, lane 2, arrowhead) was observed in the protein sample of virus-infected *H. circularisquama* cells, but it was not observed in uninfected cells (Fig. 4, lane 1). The molecular mass coincided with that of the single major protein of purified HcRNAV (Fig. 3, lane 3). By Western blotting, ABH-1 clearly recognized the ~35-kDa protein of virus-infected *H. circularisquama* cells (Fig. 4, lane 5 and 6). In addition, ~70- and ~100-kDa bands were also weakly recognized (Fig. 4, lane 5 and 6); these may correspond to a dimer and trimer of intact HcRNAV MCP as no recognition signal by ABH-1 was detected in uninfected *H. circularisquama* cells (Fig. 4, lane 4). Based on these results, ABH-1 was considered suitable for use in further experiments on HcRNAV because of its high specificity and high reactivity.

### Detection of virus-infected cells by immunofluorescence microscopy

**Fig. 5:**
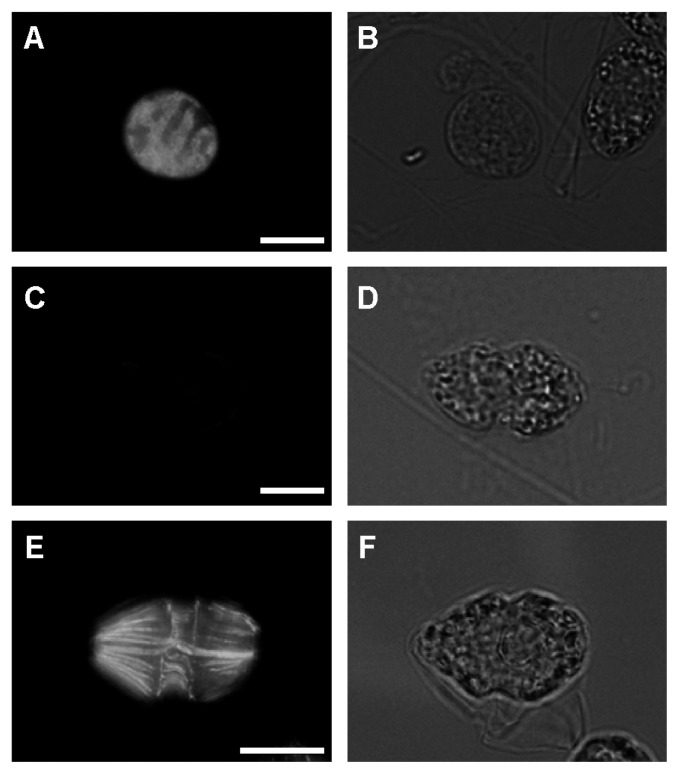
Immunofluorescence micrographs (A, C, E) and differential interference contrast images (B, D, F) of HcRNAV-infected *H. circularisquama* cells immunostained with the anti-MCP polyclonal antibody ABH-1 (A, B) and uninfected cells (C, D); the latter served as the negative control. In A, note the presence of a brightly labeled region in the cytoplasm. Uninfected cells immunostained with anti-alpha- tubulin antibody served as the positive control (E, F). Scale bars indicate 20 μm.

To confirm the ability of ABH-1 to detect HcRNAV-infected cells, immunofluorescence microscopic analysis was conducted. In these experiments, *H. circularisquama* cells treated with anti-alpha-tubulin antibody (Clone DM1A) served as the positive control, which specifically recognizes alpha-tubulin found in a wide range of organisms (3). Microtubule filaments (skeletal microtubules) (25) and transversal flagella prepared from microtubules were positively probed with the FITC-conjugated secondary antibody. Hence, this immuno-cytological method was shown to be appropriate to estimate the reactivity of ABH-1 to *H. circularisquama* cells. As a result, in the virus-inoculated *H. circularisquama* culture, a small portion of cells was probed with ABH-1, which showed green fluorescence of the FITC-labeled antibody using an indirect immuno-fluorescence technique (Fig. 5A, B). Fluorescence was dispersed throughout the entire cytoplasm, and positively probed cells were spherical. In contrast, we did not detect any cells showing green fluorescence in the uninfected culture (Fig. 5C, D), and the cells were typically spindle-shaped, as reported previously (6). The reason why only a small portion (ca. 2%) of cells was stained is unknown. One possible explanation is that HcRNAV propagation occurred only in some of the cells even in a clonal population despite the possibly of high multiplicity of infection, as was observed in a previous study on the virus resistance of *H. circularisuqama* (34).

Another question is whether ABH-1 is able to recognize intracellular HcRNAV MCP. Based on the epifluorescence photograph shown in Fig. 5A, almost all the cytoplasmic area appears positively immunostained, but with some non-uniformity. On the other hand, virus particles are usually observed as forming patches in a cell (*e.g.* Tomaru *et al.* 2004). Considering these two possibilities, it is conceivable that the intracellular region with mature virion assembly was densely stained and that with soluble-form MCP molecules was weakly stained (Fig. 5A). To verify this hypothesis and reach a rational interpretation, establishment of an immuno-electron microscopy system for HcRNAV within its host cell is now underway, which should exhibit the distribution of MCP in the host cell.

### Codon usage in *H. circularisquama* genes

In this study, codon usage for *H. circularisquama* is an important issue. The MCP gene was designed by following the deduced amino acid sequence of HcRNAV34 MCP under the assumption that translation is performed following universal codon usage in the host dinoflagellate; however, only a limited amount of data concerning the codon-usage pattern of dinoflagellates has been accumulated to date. According to some EST studies of several dinoflagellates (2, 7, 21, 22), their codon usage is predicted to be universal. In addition, in the case HcRNAV study, the amino acid sequence of the N-terminal region, independently determined by the Edman degradation method (16) and the universal deduction method coincided well with each other. Furthermore, VLPs were successfully formed by the self-assembly of recombinant MCP in this study, suggesting it had the proper 3D/4D configuration. Based on these results, we conclude that the VLPs obtained in this study were structurally equivalent to native HcRNAV, but lacked genomic ssRNA, and also the codon usage for *H. circularisquama* is probably “universal”.

### Conclusion and future perspectives

In this study, we developed a polyclonal antibody against MCP. The obtained antiserum was strongly reactive not only with bacterially expressed MCP, but also with native HcRNAV MCP. In addition, immunofluorescence analysis revealed that HcRNAV in the host cells could be detected using the antiserum. Our results demonstrate the feasibility and usefulness of the polyclonal antibody ABH-1; it would be useful to estimate the progression rate of infection in order to elucidate the HcRNAV infection process, establish an immunochemical enumeration method for HcRNAV, enumerate infection prevalence, and to identify the receptor protein to which HcRNAV adsorbs at the initial stage of infection.

## Supplementary Material
