# Supplementary material for: Establishment of a Bacterial Expression System and Immunoassay Platform for the Major Capsid Protein of HcRNAV, a Dinoflagellate-Infecting RNA Virus
Source: Microbes Environ. 2012 Oct 5;27(4):483–9. doi: 10.1264/jsme2.ME12046 (PMC4103558; doi:10.1264/jsme2.ME12046)
Supplement: Supplementary file 1 [file 27_483_s1.pdf]

	E. coli cells	Yeast cells	Incest cells	*E. coli* cells
The gene used for expression	Authentic MCP gene			Synthesized MCP gene
Plasmids construction	pET15b, pET21a, pT7, pBBR, pCold-I or pCold-GST	pHIL-D2 or pPIC9	pFastBac1 and Bacmid	pET15b, pET21a, pT7, pBBR, pCold-I or pCold-GST
Transformation of the plasmids	BL21(DE3), C41(DE3), Origami(DE3), Rosetta(DE3), Rosetta2(DE3), Origami(DE3), Rossetta-gami(DE3)<br>DH5α or JM109	*Pichia pastoris* GS115	Sf9	BL21(DE3), C41(DE3), Origami(DE3), Rosetta(DE3), Rosetta2(DE3), Origami(DE3), Rossetta-gami(DE3)<br>DH5α or JM109
Induction of the expression	IPTG and/or Low temperature	Methanol	None (constitutive)	IPTG and/or Low temperature
**Results**	Unexpressed	Unexpressed	Unexpressed	Expressed<br>(The combination of C41(DE3) and pCold-GST plasmid only)

Fig. S1. Strategy and brief results of all expressions.

Initially, we had begun to establish an overproduction system of recombinant HcRNAV MCP using the authentic nucleotide sequence in *E. coli* cells. We had used several plasmids, such as pET15b, pET21a, pT7, pBBR, pCold-I and pCold-GST, which were driven by the T7, lac and cspA promoter, respectively. The ORF region with authentic sequence encoding MCP was cloned into these plasmids and transformed into the various host cells. For the T7 and cspA promoter-derived plasmids, BL21 (DE3), C41 (DE3), Origami (DE3), Rosetta (DE3), Rosetta2 (DE3), Origami (DE3), Rossetta-gami (DE3) were used as the host cells. For the lac promoter-driving plasmid, DH5 and JM109 were used. For the small-scale culture, the transformants were grown at 37°C or 28 °C in 2 mL liquid LB or TB medium containing adequate antibiotics to an optical density of 0.6 at 600 nm. At this stage, the expression was induced by adding IPTG at a final concentration of 1 mM or 0.1 mM (and for cspA promoter, the culture temperature was also shifted to 15°C). Under all conditions, MCP was not able to express either the soluble or insoluble fractions of *E. coli* cells.

We next attempted the expression using yeast and insect cells. Briefly, for the expression in yeast, the gene fragment encoding the N-terminal His-tagged authentic ORF2 derived from pET-15b-orf2 vector was cloned into pHIL-D2 and pPIC9 vectors, and the vector constructs were integrated into the genome of P. pastoris. Expression of His-tagged ORF2 was also attempted using insect cells, in which the isolation and transfection of bacmid DNA into Sf21 cells was carried out according to the Bac-to-Bac baculovirus expression system manual (Invitrogen). These expression trials were also unsuccessful.

Finally, considering that there are many rare codons in the HcRNAV MCP nucleotide sequence, an artificial sequence to optimize codon usage for expression in *E. coli* cells was designed on the basis of the deduced amino acid sequence of HcRNAV MCP and synthesized de novo. We used the same culture conditions used for the authentic MCP gene and, only when the pCold-His6-GST-ORF2 expression vector was transformed into the *E. coli* strain C41 (DE3) was His-GST-MCP successfully overexpressed in the insoluble fraction. We emphasize that the expression of MCP was successful only using the combination of the synthesized gene and the pCold-GST plasmid.

```
                       10        20        30        40        50        60        70        80        90       100
Native MCP     ATGACCCGTCCCCTAGCTCTTACCAATGGCGGCAATACTAATGGAGGCAATAACGGCGGCTCTCGCCGCCGTCCTCCCCGACAGCGCCGCCAGGGCAGGC
               MetThrArgProLeuAlaLeuThrAsnGlyGlyAsnThrAsnGlyGlyAsnAsnGlyGlySerArgArgArgProProArgGlnArgArgGlnGlyArgA
Optimized MCP  ATGACCCGCCCACTTGCTTTAACGAATGGTGGCAATACAAATGGCGGTAATAATGGAGGCTCTCGTCGTCGCCCACCTCGTCAACGTCGTCAAGGTCGTC

                      110       120       130       140       150       160       170       180       190       200
               GCCGAAACCGGCGCAGGGGCGGTGGGGGTGGCGGCCCCCGTAACAATGCCGCTATGGTGTTGGCCCAGGGAGCTGGGTCCGTGCCCGGGATGCCTTTTGG
               rgArgAsnArgArgArgGlyGlyGlyGlyGlyGlyProArgAsnAsnAlaAlaMetValLeuAlaGlnGlyAlaGlySerValProGlyMetProPheGl
               GTCGTAACCGTCGTCGCGGTGGTGGTGGAGGCGGTCCACGTAATAATGCCGCGATGGTTCTTGCCCAAGGTGCTGGCTCTGTCCCCGGTATGCCCTTCGG

                      210       220       230       240       250       260       270       280       290       300
               CAGCTGGCCTTCGCGTAGTACAATGCGAGCCTGGGATGCCTTTCACCCTGAGCACCTTCCGCTCCCTCGGTCAGTGGGGCCCTATTGCGTGGTTCGCACG
               ySerTrpProSerArgSerThrMetArgAlaTrpAspAlaPheHisProGluHisLeuProLeuProArgSerValGlyProTyrCysValValArgThr
               TTCCTGGCCTAGTCGTTCAACAATGCGCGCTTGGGACGCTTTTCATCCAGAACATCTCCCTCTCCCCCGTAGTGTTGGCCCGTATTGTGTAGTTCGCACC

                      310       320       330       340       350       360       370       380       390       400
               AGCAGCTTGATTACGTCCAGTGACAAGGTCATGTTGTTTGCTCCCATGGTTGGCAGCGCCGGCTGCTGGCTAACGGCATGTGCGATGGGGTCCCGCACTG
               SerSerLeuIleThrSerSerAspLysValMetLeuPheAlaProMetValGlySerAlaGlyCysTrpLeuThrAlaCysAlaMetGlySerArgThrG
               AGCTCTTTAATTACCTCTAGCGATAAAGTGATGTTATTTGCGCCTATGGTTGGCTCCGCCGGTTGTTGGCTTACAGCATGTGCTATGGGATCACGTACTG

                      410       420       430       440       450       460       470       480       490       500
               AAGGCGGGGCTATTAATGGACTTGATAATACCAACGTGTACACCGTACCCTTTCCGGGGATCGCAACCACTGGTAGCAGTATCACGGTCGTGCCTGCGGC
               luGlyGlyAlaIleAsnGlyLeuAspAsnThrAsnValTyrThrValProPheProGlyIleAlaThrThrGlySerSerIleThrValValProAlaAl
               AAGGCGGCGCGATTAATGGTTTAGATAACACCAATGTTTACACCGTCCCTTTTCCTGGTATTGCCACAACCGGATCATCAATTACAGTAGTTCCCGCTGC

                      510       520       530       540       550       560       570       580       590       600
               GTTGTCAGTCCAGGTTATGAACCCCAACCCACTTATGTCCACCACTGGAATCTTTGGTGGCACAGTTTCACACACCCAGCTTAATCTTGCAGGGCGTACA
               aLeuSerValGlnValMetAsnProAsnProLeuMetSerThrThrGlyIlePheGlyGlyThrValSerHisThrGlnLeuAsnLeuAlaGlyArgThr
               GTTAAGTGTTCAAGTTATGAATCCCAACCCTCTTATGTCGACCACTGGTATTTTCGGCGGTACAGTTTCTCATACTCAATTAAACCTTGCAGGCCGTACC

                      610       620       630       640       650       660       670       680       690       700
               GAGACCTGGAATGATTTCTCTATGGAGGTGATTTCTTTTATGAGGCCGCGGCTTATGTCGGCTGGGAAGCTTGCCCTGCGCGGAGTCCAGGGAGATAGCT
               GluThrTrpAsnAspPheSerMetGluValIleSerPheMetArgProArgLeuMetSerAlaGlyLysLeuAlaLeuArgGlyValGlnGlyAspSerT
               GAAACCTGGAATGATTTTAGCATGGAAGTGATCTCGTTTATGCGCCCACGTCTGATGTCCGCTGGTAAATTAGCGTTGCGTGGCGTACAGGGTGATTCCT

                      710       720       730       740       750       760       770       780       790       800
               ACCCCCTTAATATGTCCGCGTTGTCCAACTTCAATTGTCTTAGCGACGTGGCAGAAGGCAAGCTTTCGTGGACTGATTCCAGCGGGCATTACCCGGCTGG
               yrProLeuAsnMetSerAlaLeuSerAsnPheAsnCysLeuSerAspValAlaGluGlyLysLeuSerTrpThrAspSerSerGlyHisTyrProAlaGl
               ATCCGCTTAACATGTCCGCCCTGTCTAACTTCAATTGTTTAAGCGATGTTGCAGAAGGCAAATTATCATGGACGGATTCATCGGGACATTATCCGGCTGG

                      810       820       830       840       850       860       870       880       890       900
               TTTGGCGCCGTTAGTCTTTGTGAACGAGGCAAAGCAAACAATGAACTATCTGGTGTCCGTCGAATGGCGCGTCCGGTTTGACATCGGCAACCCTGCTGTT
               yLeuAlaProLeuValPheValAsnGluAlaLysGlnThrMetAsnTyrLeuValSerValGluTrpArgValArgPheAspIleGlyAsnProAlaVal
               CTTAGCCCCACTTGTATTTGTAAACGAAGCAAAACAAACTATGAATTATCTCGTTTCAGTTGAGTGGCGTGTTCGTTTTGACATTGGAAATCCTGCAGTT

                      910       920       930       940       950       960       970       980       990      1000
               GCCGCCCAACGCCACCACGGCATTACGCCGGAGTGGAAGTGGGATGATATGATTAAGACTGCGATTGCTCGCGGTCACGGTATTATGGATATCGCTGAGC
               AlaAlaGlnArgHisHisGlyIleThrProGluTrpLysTrpAspAspMetIleLysThrAlaIleAlaArgGlyHisGlyIleMetAspIleAlaGluA
               GCGGCCCAACGGCATCATGGAATTACACCTGAATGGAAATGGGACGATATGATCAAAACCGCAATCGCACGTGGTCATGGTATTATGGATATTGCAGAAC

                     1010      1020      1030      1040      1050      1060      1070      1080
               GTGTCGCCAACGCGGGCTCGTTTGTCGCAAACGCTGCGATTGCGGCCCGCAGAGCTATGCCAGCATTGATGGCTGCTTAA
               rgValAlaAsnAlaGlySerPheValAlaAsnAlaAlaIleAlaAlaArgArgAlaMetProAlaLeuMetAlaAla***
               GCGTAGCGAACGCTGGTTCTTTTGTCGCCAATGCAGCAATTGCAGCTCGTCGTGCTATGCCTGCCCTGATGGCTGCCTAA
```

Fig. S2. Comparison of the nucleotide sequence of native HcRNAV MCP gene (upper line), its deduced amino acid sequence following universal codon usage (middle line), and nucleotide sequence of the artificially synthesized gene optimized for expression in *E. coli* (bottom line). Codons differing between upper and bottom lines are highlighted in color.

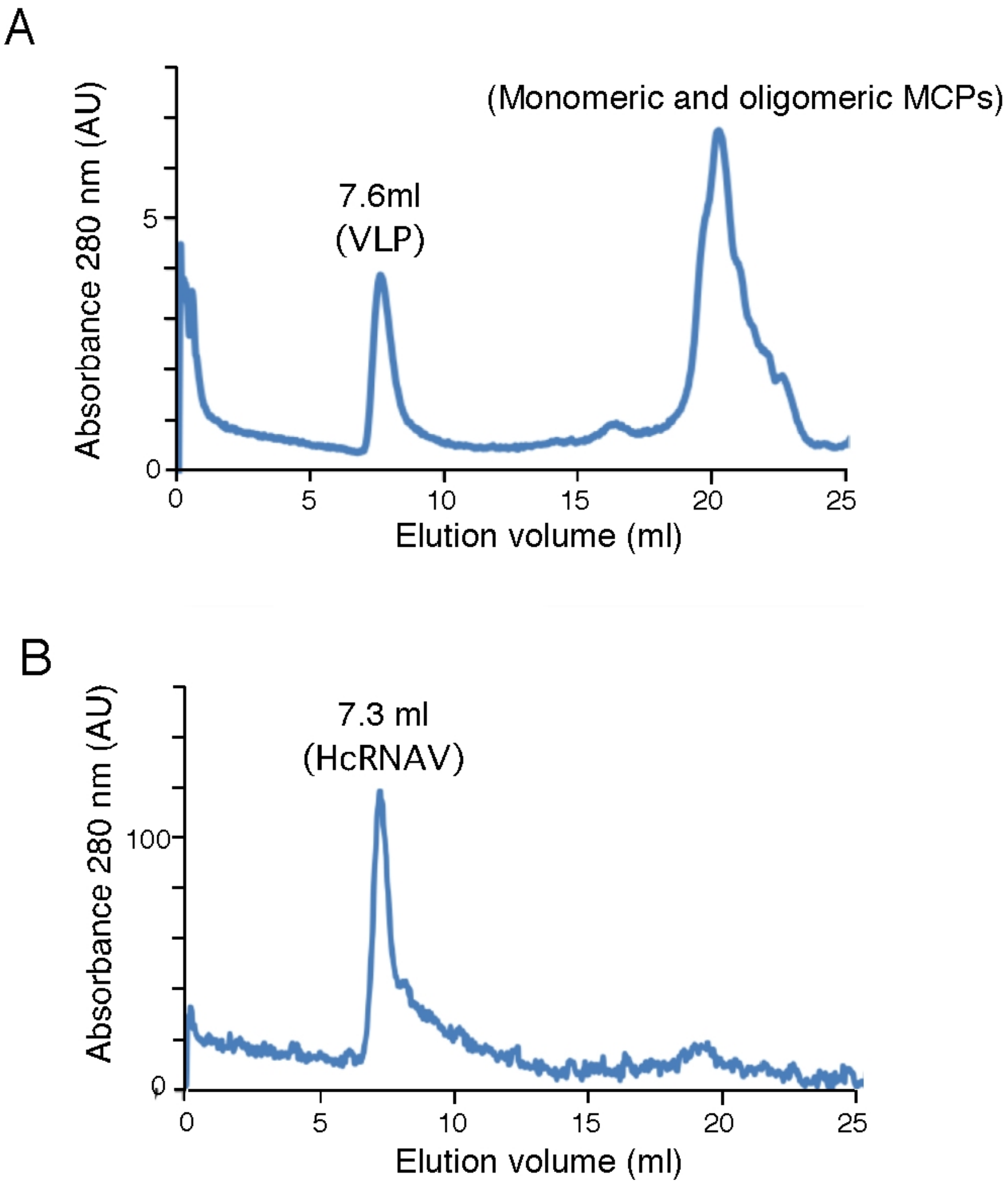

Fig. S3. Gel-filtration analyses of VLP and HcRNAV. (A) Chromatogram of VLP. Recombinant MCP was reconstituted by the dilution method and then subjected to gel-filtration (Superose 6 10/300 GL; GE Healthcare) chromatography. (B) Chromatogram of purified HcRNAV. HcRNAV was collected from the HcRNAV-infected *H. circularisquama* culture by the polyethylene-glycol precipitation method, and then gel-filtration analysis was performed under the same conditions as for VLP.
